# Long-term follow-up of mental health, health-related quality of life and associations with motor skills in young adults born preterm with very low birth weight

**DOI:** 10.1186/s12955-016-0458-y

**Published:** 2016-04-07

**Authors:** Ingrid Marie Husby, Kaia Mølbach-Thellefsen Stray, Alexander Olsen, Stian Lydersen, Marit Sæbø Indredavik, Ann-Mari Brubakk, Jon Skranes, Kari Anne I. Evensen

**Affiliations:** Department of Laboratory Medicine, Children’s and Women’s Health, Norwegian University of Science and Technology, Trondheim, Norway; Stamina Helse, Lillestrøm, Norway; Department of Psychology, Norwegian University of Science and Technology, Trondheim, Norway; Department of Physical Medicine and Rehabilitation, St. Olavs Hospital, Trondheim University Hospital, Trondheim, Norway; MI Lab and Department of Circulation and Medical Imaging, Norwegian University of Science and Technology, Trondheim, Norway; Regional Centre for Child and Youth Mental Health and Child Welfare, Faculty of Medicine, Norwegian University of Science and Technology, Trondheim, Norway; Department of Child and Adolescent Psychiatry, St. Olavs Hospital, Trondheim University Hospital, Trondheim, Norway; Department of Pediatrics, Sørlandet Hospital, Arendal, Norway; Department of Public Health and General Practice, Norwegian University of Science and Technology, Trondheim, Norway; Department of Physiotherapy, Trondheim Municipality, Trondheim, Norway

**Keywords:** Prematurity, Very low birth weight, Long-term outcome, Mental health, Health-related quality of life, Young adulthood, ASEBA, BDI, SF-36, Motor skills

## Abstract

**Background:**

Being born with very low birth weight (VLBW: ≤1500 g) is related to long-term disability and neurodevelopmental problems, possibly affecting mental health and health-related quality of life (HRQoL). However, studies in young adulthood yield mixed findings. The aim of this study was to examine mental health and HRQoL at 23 years, including changes from 20 to 23 years and associations with motor skills in VLBW young adults compared with controls.

**Methods:**

In a geographically based follow-up study, 35 VLBW and 37 term-born young adults were assessed at 23 years by using Achenbach Adult Self-Report (ASR), Short Form 36 Health Survey (SF-36), Beck Depression Inventory (BDI) and various motor tests. The ASR and SF-36 were also used at 20 years. Longitudinal changes in ASR and SF-36 from 20 to 23 years were analysed by linear mixed models and associations with motor skills at 23 years by linear regression.

**Results:**

At 23 years, total ASR score was 38.6 (SD: 21.7) in the VLBW group compared with 29.0 (SD: 18.6) in the control group (*p* = 0.048). VLBW participants had higher scores for attention problems, internalizing problems and critical items, and they reported to drink less alcohol than controls. BDI total score did not differ between groups. On SF-36, VLBW participants reported significantly poorer physical and social functioning, more role-limitations due to physical and emotional problems, more bodily pain and lower physical and mental component summaries than controls. In the VLBW group, total ASR score increased by 9.0 (95 % CI: 3.3 to 14.7) points from 20 to 23 years (*p* = 0.009 vs controls), physical and mental component summaries of SF-36 decreased by 2.9 (95 % CI: -4.8 to -1.1) and 4.4 (95 % CI: -7.1 to -1.7) points, respectively (*p* = 0.012 and *p* = 0.022 vs controls). Among VLBW participants, more mental health problems and lower physical and mental HRQoL were associated with poorer motor skills at 23 years.

**Conclusions:**

VLBW young adults reported poorer and declining mental health and HRQoL in the transitional phase into adulthood. They seemed to have a cautious lifestyle with more internalizing problems and less alcohol use. The associations of mental health problems and HRQoL with motor skills are likely to reflect a shared aetiology.

**Electronic supplementary material:**

The online version of this article (doi:10.1186/s12955-016-0458-y) contains supplementary material, which is available to authorized users.

## Background

As neonatal medicine has been improving for the last decades, more very low birth weight (VLBW; birth weight ≤1500 g) infants survive. The preterm brain is especially vulnerable to injury and developmental disturbances [1], increasing the risk of later neurodevelopmental problems [2, 3]. This may have an impact on mental health and health-related quality of life (HRQoL); however, studies on long-term effects of VLBW into adulthood are sparse and yield mixed findings.

Children and adolescents born preterm with VLBW are reported to have more mental health problems than full-term controls, with an increased occurrence of attention deficits, internalizing symptoms and social problems in particular [2, 4]. There is an important transitional phase from adolescence to adulthood involving increasing demands on independency, education and adult roles [5], which may stress the underlying neuroimpairments in VLBW individuals. Indeed, preterm birth is shown to have an adverse effect on educational attainment, income and establishment of a family [6], and mental health problems tend to persist or even increase into young adulthood [7–9].

The concept of HRQoL refers to the impact of health conditions on a person’s total well-being, including psychological, social, and physical aspects [10]. Measuring HRQoL gives valuable insight into the person’s perception of his or her own health status, complementing more objectively collected data, and should therefore be addressed when assessing long-term consequences of VLBW. Although self-reports of HRQoL in VLBW children and adolescents seem to be similar to their normal birth weight peers, parent-reports are typically lower [11]. Some studies of VLBW young adults have revealed lower HRQoL based on societal standards [12] and lower scores on HRQoL domains of mental health [13] and physical functioning [14, 15]. Other studies report similar HRQoL [14, 16, 17] and well-being [18] for VLBW young adults compared with controls. Longitudinal studies on changes of HRQoL in VLBW populations are sparse, but receive growing attention [11, 19].

Developmental disturbances in the preterm brain are global and likely to affect both mental health and other areas of neurodevelopment, such as motor problems. Poorer fine and gross motor skills are prevalent in childhood, adolescence and young adulthood in VLBW individuals [3, 20]. Both among adults with normal birth weight and <1000 g, self-reported childhood coordination problems have been associated with elevated levels of inattention and symptoms of anxiety and depression [21]. Lower quality of life has been reported among adults with developmental coordination disorder [22]. However, no previous studies have investigated associations of mental health and HRQoL with motor skills in VLBW young adults. As motor skills are often assessed in childhood, and we have previously reported stability of motor problems from early childhood to young adulthood [20, 23], it may be possible to identify children at risk for later mental health problems and low HRQoL.

In this study, we aimed to investigate the effects of VLBW on mental health and HRQoL in young adults at 23 years of age, including changes from 20 to 23 years and whether mental health and HRQoL were associated with motor skills. We hypothesized that VLBW young adults at age 23 would have more mental health problems and lower HRQoL compared with controls. Due to increased demands following the transition to adulthood, we predicted a decrease in mental health and HRQoL from 20 to 23 years in the VLBW group. Based on previous findings, we hypothesized that more mental health problems and lower physical HRQoL would be associated with poorer motor skills at age 23.

## Methods

### Study design

This is a geographically based follow-up study of young adults born preterm with VLBW and a control group born at term with normal birth weight at 23 years. The VLBW children were born in 1986-1988 and admitted to the Neonatal Intensive Care Unit (NICU) at St. Olavs Hospital, Trondheim University Hospital, Norway. The control children were born in the same period to mothers living in the Trondheim region (total population approximately 135.000), recruited from a 10 % random sample of women selected for follow-up during pregnancy in a multicentre study on causes and consequences of intrauterine growth restriction [24]. Both groups had previously participated in the study with evaluations at 1, 5, 14 and 20 years of age [13, 23, 25]. At 23 years, we aimed to include all VLBW participants from the 14-year follow-up and a selection of control participants, matched by age and sex, due to lack of resources. Self-report questionnaires on mental health and HRQoL were included as part of a large assessment battery, which also covered executive tests, motor tests and cerebral magnetic resonance imaging. Data on mental health and HRQoL were also available from the 20-year follow-up, and results have been published previously [13].

### Study groups

**Fig. 1:**
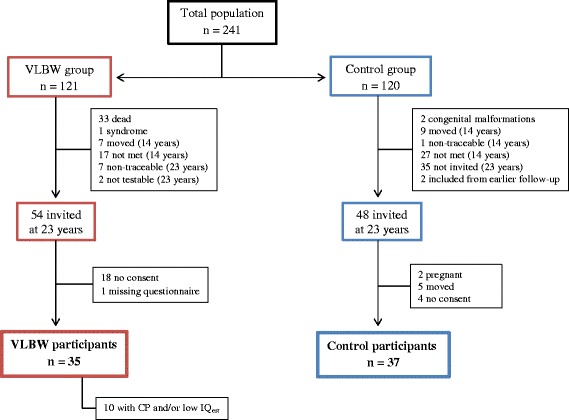
Flow chart of the study groups. *VLBW* = very low birth weight, *CP* = cerebral palsy, *IQ*_*est*_ = estimated intelligence quotient

Figure 1 shows a flow chart of the study groups.

#### VLBW group

At 14 years, 63 VLBW adolescents with birth weight ≤1500 g had been examined. At 23 years, seven of these were non-traceable and two were excluded due to severe bilateral spastic cerebral palsy (CP) of Gross Motor Function Classification System (GMFCS) level V [26]. Thus, we contacted 54 VLBW young adults, whereof 18 (33 %) did not consent and one did not fill out the questionnaires, leaving 35 participants (14 males and 21 females) in the VLBW group (Fig. 1). At 20 years, mental health and HRQoL had been assessed in 52 VLBW participants, whereof nine were excluded from this paper due to missing data on estimated intelligence quotient (IQ_est_). Hence, 43 VLBW participants with mean age 19.7 (0.8) were included in longitudinal analyses at 20 years, whereof 29 were examined at both time-points.

#### Control group

At 14 years, 81 control adolescents born at term with birth weight ≥10^th^ percentile for gestational age had been examined. At 23 years, we contacted 48 controls matched to the VLBW participants by age and sex, of which 46 were examined at the 14-year follow-up. Two were not testable due to pregnancy, five had moved and four (9 %) did not consent, leaving 37 participants (15 males and 22 females) in the control group (Fig. 1). At 20 years, mental health and HRQoL were examined for 77 controls with mean age 19.7 (0.5), whereof 31 were examined at both time-points.

#### Non-participants

In the VLBW group, there were no significant differences between those who participated and those who did not consent to participation at age 23 regarding perinatal data (data not shown). Parental socioeconomic status (SES) at 14 years was 3.5 (1.1) among participants compared with 2.8 (1.3) among non-participants (*p* = 0.045, Mann–Whitney *U* test). In the control group, there were no significant differences between those who participated and those who did not consent to participation or were not contacted from previous follow-up (data not shown). Furthermore, there were no significant differences in motor skills at 14 years [20] or summary scores for mental health or HRQoL at 20 years between participants and non-participants at the 23-year follow-up in either group (data not shown).

### Data collection

#### Clinical characteristics

Perinatal data included birth weight, gestational age, head circumference, Apgar scores, days in NICU, days on mechanical ventilator, intraventricular haemorrhage status and maternal age. CP was diagnosed in childhood, and classified as spastic hemiplegia (unilateral spastic CP), diplegia (bilateral spastic CP, mainly involvement of lower extremities) and quadriplegia (bilateral spastic CP with involvement of all extremities). Functional level was assessed according to the GMFCS [26]. At 14 years, IQ_est_ was calculated at using two subscales of Wechsler Intelligence Scales for Children, third edition; vocabulary and block design [27]. “Low IQ_est_” was defined as a score more than two standard deviations below the mean in the control group (IQ_est_ <69). Parental SES was calculated according to Hollingshead’s two factor index of social position [28], rated from 1 (lowest) to 5 (highest) based on a combination of parents’ education and occupation at the 14-year follow-up.

#### Follow-up at 23 years

The self-report questionnaires and the motor tests were carried out at the same day and at the same location. For one participant with unilateral spastic CP and low IQ_est_, the self-report was carried out as an interview by one of the researchers.

#### Mental health: Adult Self-Report (ASR)

Mental health was measured by the Adult Self-Report (ASR) [29], which is part of the Achenbach System of Empirically Based Assessment (ASEBA), a worldwide used instrument shown to be reliable and valid [29]. An authorized Norwegian translation of the ASEBA was applied. The ASR (age range 18–59) comprises 120 problem items (scored 0–2), yielding eight syndrome scales (score range): anxious/depressed (0–36), withdrawn (0–18), somatic complaints (0–24), thought problems (0–20), attention problems (0–30), aggressive behaviour (0–30), rule-breaking behaviour (0–28) and intrusive (0–12). The first three scales comprise the composite scale for internalizing problems whereas the last three comprise the composite scale for externalizing problems. The sum of all problem items yields a total problems score (range: 0–240). A score for critical items is made by summing the scores on 19 problem items evaluated to be the most clinically relevant psychiatric symptoms. Raw scores are used, and higher scores indicate more problems. In addition, the ASR includes items on adaptive functioning, of which we used scales for friends, family and substance use. The scale for friends yield a total score based on number of close friends, frequency of contact with friends, getting along with friends and visits from friends, each scored 0–3, with a maximum of 12 points. The scale for family relations yield a mean score based on self-perceived quality of relation with parents and/or siblings compared with others, each scored 0–2. Higher scores for friends and family indicate better adaptive functioning. Substance use includes tobacco (number of cigarettes smoked daily), alcohol (number of days being drunk last 6 months) and drugs (number of days using drugs last 6 months). T-scores are recommended when having extreme outliers [29], and are also used to calculate a mean substance use scale. One VLBW participant did not complete the ASR at 23 years. For one VLBW participant, reliable data on alcohol use was missing at age 20 and excluded in the analysis for longitudinal change in alcohol use.

#### Mental health: Beck Depression Inventory (BDI), Version IA

The BDI [30] is among the most used self-rating scales for measuring depression, and has high validity in differentiating between depressed and non-depressed participants [31]. The BDI (age ≥13) consists of 21 questions measuring symptoms and severity of depression during the past week, including today. Values range from 0 to 3 and are summed into a total score, where a score ≥21 indicates depression in the general population [30]. One VLBW and two control participants did not complete the BDI at 23 years. The BDI was not used at 20 years.

#### Health-related quality of life: Short Form 36 Health Survey (SF-36), Version 1.0

The SF-36 is a generic measure of HRQoL with high reliability and it has been validated for use across a range of health care professions, settings and patients [32], also for the Norwegian translation applied in this study [33, 34]. The questionnaire comprises 36 items across eight domains: physical functioning, role limitations due to physical problems (role-physical), bodily pain, general health, vitality, social functioning, role limitations due to emotional problems (role-emotional) and mental health. Raw scores are transformed into an aggregate percentage score for each domain ranging from 0 to 100 %, where higher percentage indicate favourable health outcome and higher level of functioning. The two domains role-physical and role-emotional have dichotomised response choices, while the other domains have a Likert-type response with three to six choices. The recall period is 4 weeks, except for physical functioning and general health, which address current status. Three of the domains (physical functioning, role-physical and bodily pain) contribute mainly to a physical component summary, while three other domains (social functioning, role-emotional and mental health) contribute mainly to a mental component summary. Three of the domains (general health, vitality and social functioning) have noteworthy correlations with both components [32]. We applied an oblique model for calculating the component summaries after recommendation from Hann and Reeves [35]. We used a Certified Scoring Software 4.0™ to score the questionnaire and the official calculator at the home page of SF-36 to calculate the component summaries [36] based on Norwegian normative data [37] with average of 50 points and a standard deviation of 10 points. One control had missing domain scores for vitality and mental health because of too many missing items, and we could therefore not calculate component summaries for this participant. Two controls did not complete the SF-36 at 23 years.

#### Motor examination

Motor skills were examined by using four different motor tests, described in detail in a previous paper on motor skills in the same study population [20]. The Grooved Pegboard (GP) [38] is a manipulative dexterity test giving a score for each hand separately, which we calculated into a mean score for both hands. The Trail Making Test-5 (TMT-5) is one of five subtests in the standardized Delis-Kaplan Executive Function System [39] measuring motor speed of the dominant hand. The Movement Assessment Battery for Children-2 (Movement ABC-2) [40] consists of three components (manual dexterity, aiming and catching, and balance) yielding a total score. The High-Level Mobility Assessment Tool (HiMAT) [41] assesses 13 gross motor items (walk, walk backwards, walk on toes, walk over obstacle, run, skip, hop forward, bound, and walk up/down stairs) yielding a total score. For the GP and TMT-5, higher scores indicate poorer function and for Movement ABC-2 and HiMAT, higher scores indicate better function.

### Ethical approval and consent

The project complies with the principles of the Declaration of Helsinki and was approved by the Regional Committee for Medical and Health Research Ethics in Central Norway. Written informed consent was obtained from all participants.

### Statistical analyses

Student’s *t*-test was used for approximately normally distributed data; else the Mann–Whitney *U* test was applied. Descriptive statistics are reported as mean (SD) where relevant. To limit the number of statistical tests, we only included the summary scores of ASR (critical items, internalizing, externalizing and total problems) and SF-36 (physical and mental component summaries) for longitudinal changes and associations with motor skills. Linear mixed models were used to analyse changes in summary scores from 20 to 23 years. Summary scores were entered separately as dependent variables, whereas age, group and the interaction age x group were entered as independent variables in analyses, both unadjusted and adjusted for sex. Linear regression was applied to explore associations of mental health and HRQoL with motor skills. Summary scores of ASR and SF-36 were entered separately as dependent variables, whereas motor test, group and the interaction motor test x group were entered as independent variables. The interaction terms were added to test if the effect of time or motor skills were different in VLBW and control participants. Normality was judged by visual inspection of Q-Q plots of the residuals. If outliers were observed, sensitivity analyses excluding the outlier were carried out. Two-sided *p*-values <0.05 were considered statistically significant. Analyses were performed both including and excluding participants with CP and/or low IQ_est_. SPSS 22.0 was used for data analyses.

## Results

### Clinical characteristics

Clinical characteristics are shown in Table 1. Age at current follow-up was 22.5 (0.7) years in the VLBW group and 22.7 (0.6) years in the control group (*p* = 0.234, Student’s *t*-test). Maternal age at birth and parental SES at age 14 did not differ between groups (*p* = 0.165, Student’s *t*-test and *p* = 0.580, Mann–Whitney *U* test, respectively). Four VLBW participants had CP; one female had unilateral spastic CP of GMFCS level I and three males had bilateral spastic CP with GMFCS level I, II and IV. One control and eight VLBW participants had low IQ_est_ at age 14, whereof two had CP.

**Table 1:** Clinical characteristics

	VLBW (*n* = 35)		Control (*n* = 37)	
	Mean	(SD)	Mean	(SD)
Birth weight (g)	1198	(254)	3608	(361)
Gestational age (weeks)	29.0	(2.7)	39.4	(1.1)
Birth head circumference (cm)^a^	26.9	(2.4)	35.2	(1.2)
Apgar score after 1 min^b^	6.8	(1.9)	8.9	(0.5)
Apgar score after 5 min^c^	8.4	(1.7)	9.7	(1.5)
Maternal age at birth (years)	28.4	(5.6)	30.1	(4.5)
Parental SES (at 14 years)^c^	3.5	(1.1)	3.7	(1.1)
Age at current follow-up	22.5	(0.7)	22.7	(0.6)
	Median	(Range)	Median	(Range)
Stay in NICU (days)^d^	63	(25–386)	0	(0–9)
Mechanical ventilation (days)^d^	1	(0–63)	-	
	n	(%)	n	(%)
Intraventricular haemorrhage^d^	3	(9)	-	
Grade I-II	2			
Grade IV	1			
Cerebral palsy	4	(11)	0	
GMFCS level I-II	3			
GMFCS level IV	1			
Low IQ_est_	8	(23)^e^	1	(3)

^a^Data missing for eight VLBW and three control participants

^b^Data missing for three control participants

^c^Data missing for two control participants

^d^Data missing for one VLBW participant

^e^Two VLBW participants with low IQ_est_ had cerebral palsy

*VLBW* = very low birth weight, *SES* = socioeconomic status, *NICU* = neonatal intensive care unit, *GMFCS* = Gross Motor Function Classification System, *IQ*_*est*_ = estimated intelligence quotient

Group differences for maternal age, SES and age at follow-up were non-significant

### Mental health: Adult Self-Report (ASR) at 23 years

The results from the ASR are shown in Table 2. The VLBW group had significantly higher scores (indicating more problems) than the control group for the scales attention problems, internalizing problems, total problems and critical items. Aggressive behaviour was also higher in the VLBW group when we excluded one outlier in the control group (*p* = 0.032, Student’s *t*-test). The VLBW group had significantly lower alcohol use than controls. For the scales alcohol, tobacco and drugs, results did not change using T-scores (data not shown). When we excluded VLBW participants with CP and/or low IQ_est_, mean scores were still higher than for controls on nearly all scales, but only the scales critical items and alcohol use showed a significant group difference (Table 2).

**Table 2:** Results of the *ASEBA Adult Self-Report* at 23 years

	VLBW (*n* = 34)			VLBW (*n* = 24) without CP and/or low IQ_est_			Control (*n* = 37)	
	Mean	(SD)	*p*-value vs control	Mean	(SD)	*p*-value vs control	Mean	(SD)
*Syndrome scales*								
Anxious/depressed	7.0	(6.1)	0.096	7.3	(6.2)	0.095	4.6	(5.7)
Withdrawn	2.4	(3.0)	0.325	2.1	(2.6)	0.669	1.8	(2.3)
Somatic complaints	3.4	(3.4)	0.081	3.0	(2.7)	0.220	2.2	(1.9)
Thought problems	1.8	(1.8)	0.371	1.5	(1.6)	0.861	1.4	(1.8)
Attention problems	7.7	(4.6)	0.013	7.0	(4.3)	0.081	5.2	(3.5)
Aggressive behaviour	3.8	(3.3)	0.205	3.4	(3.3)	0.447	2.7	(3.7)
Rule-breaking behaviour	2.9	(3.1)	0.188	3.0	(3.6)	0.243	2.0	(2.3)
Intrusive	1.7	(1.3)	0.544	1.5	(1.4)	0.386	1.9	(2.0)
Critical items	4.6	(3.4)	0.013	4.6	(3.5)	0.037	2.8	(2.7)
Internalizing problems	12.9	(9.4)	0.042	12.3	(9.1)	0.098	8.6	(7.7)
Externalizing problems	8.3	(6.0)	0.232	8.0	(6.6)	0.412	6.6	(5.7)
Total problems	38.6	(21 .7)	0.048	36.3	(22.2)	0.173	29.0	(18.6)
*Adaptive functioning*								
Friends	10.0	(2.1)	0.059	10.2	(2.0)	0.170	10.8	(1.4)
Family	1.5	(0.4)	0.756	1.5	(0.4)	0.955	1.5	(0.4)
*Substance use*								
Tobacco	3.3	(4.8)	0.688	3.2	(5.2)	0.673	3.9	(6.3)
Alcohol^a^	5.5	(5.0)	0.008	6.3	(5.5)	0.036	10.6	(9.8)
Drugs	3.7	(17.3)	0.359	4.9	(20.5)	0.366	1.0	(4.2)
Mean substance use^a^	54.1	(2.7)	0.067	54.2	(2.6)	0.119	55.5	(3.3)

^a^Data missing for one VLBW and two control participants

Raw scores are given for all scales, except mean substance use which is given as T-score. Higher scores indicate more problems on syndrome scales and substance use, while higher scores on adaptive functioning indicate better functioning

*ASEBA* = Achenbach System of Empirically Based Assessment, *VLBW* = very low birth weight, *CP* = cerebral palsy, *IQ*_*est*_ = estimated intelligence quotient

Analyses performed with Students *t*-test

### Mental health: Beck Depression Inventory (BDI) at 23 years

Mean total BDI score was 3.3 (5.0) in the VLBW group and 4.0 (6.5) in the control group (*p* = 0.796, Mann–Whitney *U* test). Results were similar when we excluded VLBW participants with CP and/or low IQ_est_. One VLBW participant with low IQ_est_ and one control were clinically depressed according to a cut-off ≥21.

### Health-related quality of life: Short Form 36 Health Survey (SF-36) at 23 years

The results from the SF-36 are shown in Table 3. The VLBW group had significantly lower scores than controls on the physical and mental component summaries and five of the eight domains: physical functioning, role-physical, bodily pain, social functioning and role-emotional. When we excluded VLBW participants with CP and/or low IQ_est_, mean scores were still lower than for controls, although mean differences between groups were reduced and no longer significant (Table 3).

**Table 3:** Results of the *Short Form 36 Health Survey* at 23 years

	VLBW (*n* = 35)			VLBW (*n* = 25) without CP and/or low IQ_est_			Control (*n* = 35)	
	Mean	(SD)	*p*-value vs control	Mean	(SD)	*p*-value vs control	Mean	(SD)
*Domains*								
Physical functioning	90.4	(13.6)	0.018	94.6	(8.3)	0.286	96.6	(5.9)
Role-physical	80.0	(30.8)	0.005	86.0	(24.0)	0.051	96.4	(10.7)
Bodily pain	68.7	(28.3)	0.022	74.5	(23.8)	0.193	82.0	(18.3)
General health	72.1	(18.9)	0.260	70.5	(18.5)	0.477	66.8	(20.0)
Vitality^a^	49.2	(14.2)	0.091	50.5	(13.7)	0.227	54.9	(13.2)
Social functioning	86.1	(16.5)	0.025	88.5	(13.0)	0.099	94.3	(13.3)
Role-emotional	78.1	(33.3)	0.012	82.7	(29.1)	0.069	95.2	(20.0)
Mental health^a^	70.6	(16.8)	0.067	73.4	(14.1)	0.272	77.4	(13.2)
Physical component summary^a^	47.8	(5.9)	0.009	49.4	(5.2)	0.160	51.2	(4.1)
Mental component summary^a^	45.2	(8.4)	0.013	46.6	(7.4)	0.087	49.7	(6.4)

^a^Data missing for one control participant

Domain scores are given in percentage (range 0–100) and higher scores indicate better health-related quality of life

Component summaries are given as T-scores with average of 50 points and a standard deviation of 10 points

*VLBW* = very low birth weight, *CP* = cerebral palsy, *IQ*_*est*_ = estimated intelligence quotient

Analyses performed with Students *t*-test

### Changes in mental health and health-related quality of life from 20 to 23 years

**Fig. 2:**
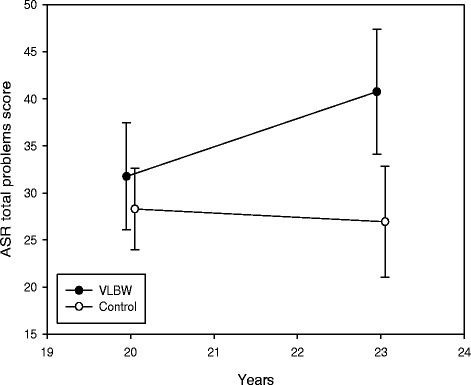
Adult Self-Report (ASR) total problems score with 95 % confidence interval at 20 and 23 years. Raw scores are given. A higher score indicates more mental health problems. *VLBW* = very low birth weight

**Fig. 3:**
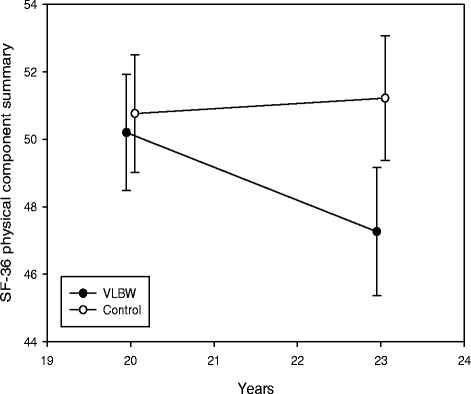
Short Form 36 Health Survey (SF-36) physical component summary with 95 % confidence interval at 20 and 23 years. T-scores are given. A higher score indicates higher physical health-related quality of life. *VLBW* = very low birth weight

**Fig. 4:**
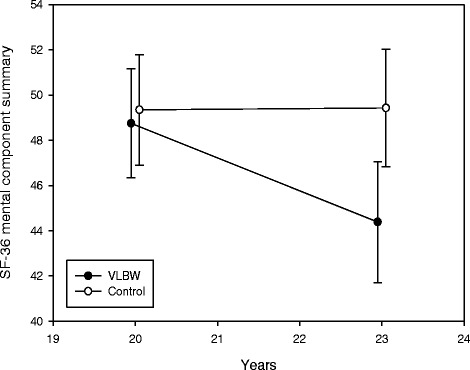
Short Form 36 Health Survey (SF-36) mental component summary with 95 % confidence interval at 20 and 23 years. T-scores are given. A higher score indicates higher mental health-related quality of life. *VLBW* = very low birth weight

Longitudinal changes in mental health and HRQoL from 20 to 23 years are shown in Table 4, visualized for ASR total problems score in Fig. 2, and physical and mental component summaries of SF-36 in Figs. 3 and 4, respectively. There were significant between-group differences with time for internalizing and total problems. In the VLBW group, ASR scores increased significantly with time (indicating more mental health problems) for internalizing, externalizing and total problems. There were significant between-group differences with time for the physical and mental component summaries. In the VLBW group, there was a significant reduction in scores with time (indicating lower HRQoL) for both the physical and mental component summaries. There were no significant changes in the control group. Adjusting for sex in the analyses did not affect the longitudinal changes (data not shown). Results for ASR were essentially the same when we excluded VLBW participants with CP and/or low IQ_est_, but for SF-36 only the mental component summary reached a statistical significant reduction with time (data not shown).

**Table 4:** Estimated changes from 20 to 23 years in scores for *ASEBA Adult Self-Report* and *Short Form 36 Health Survey*

	VLBW (*n* = 49)			Control (*n* = 83)			
	B	(95 % CI)	*p*-value	B	(95 % CI)	*p*-value	*p*-value (age x group)^b^
*Adult Self-Report*							
Critical items	0.73	(-0.47 to 1.93)	0.228	−0.38	(-1.42 to 0.66)	0.470	0.167
Internalizing problems	4.94	(1.63 to 6.24)	0.001	0.40	(-1.67 to 2.46)	0.704	0.025
Externalizing problems	1.87	(0.02 to 3.71)	0.047	−0.45	(-2.08 to 1.18)	0.583	0.064
Total problems	9.00	(3.30 to 14.71)	0.002	−1.35	(-6.46 to 3.76)	0.600	0.009
*Short Form 36 Health Survey* ^*a*^							
Physical component summary	−2.94	(-4.83 to -1.06)	0.003	0.46	(-1.39 to 2.30)	0.623	0.012
Mental component summary	−4.37	(-7.07 to -1.67)	0.002	0.08	(-2.57 to 2.73)	0.952	0.022

^a^Data missing for one control participant at both time-points

^b^p-value for between-group differences in longitudinal changes from 20 to 23 years

*ASEBA* = Achenbach System of Empirically Based Assessment, *VLBW* = very low birth weight, *CI* = confidence interval

Regression coefficient B per 3 years, in a linear mixed models with scores as dependent variables, and age, group and age x group as independent variables

### Associations with motor skills at 23 years

For the ASR, more internalizing and total problems were associated with lower motor speed on the TMT-5 (B = 0.41; 95 % CI: 0.14 to 0.67; *p* = 0.004 and B = 0.75; 95 % CI: 0.10 to 1.39; *p* = 0.024, respectively) in the VLBW group. Results were no longer significant when we excluded VLBW participants with CP and/or low IQ_est_ (data not shown). There were no significant associations of externalizing problems and critical items with motor skills.

Associations of physical and mental component summaries of SF-36 with motor skills are shown in Table 5. There was a significant between-group difference for the association of physical component summary with TMT-5. In the VLBW group, lower physical and mental component summaries were associated with poorer performance on all motor tests. When we excluded VLBW participants with CP and/or low IQ_est_, lower physical component summary was still associated with poorer performance on TMT-5 and HiMAT, while lower mental component summary was only associated with poorer performance on TMT-5 (data not shown).

**Table 5:** Associations of *Short Form 36 Health Survey* physical and mental component summaries with motor skills at 23 years

	VLBW (*n* = 35)			Control (*n* = 34)			
	B	(95 % CI)	*p*-value	B	(95 % CI)	*p*-value	*p*-value (motor test x group)^e^
*Physical component summary*							
Grooved Pegboard^a,b^	−0.09	(-0.16 to -0.02)	0.010	−0.05	(-0.23 to 0.13)	0.596	0.679
Trail Making Test-5^a^	−0.31	(-0.46 to -0.16)	0.000	0.02	(-0.25 to 0.30)	0.862	0.038
MABC-2 total score^c^	0.13	(0.04 to 0.21)	0.003	0.15	(-0.04 to 0.33)	0.125	0.847
HiMAT total score^c,d^	0.39	(0.18 to 0.59)	0.000	0.06	(-0.46 to 0.58)	0.823	0.244
*Mental component summary*							
Grooved Pegboard^a,b^	−0.15	(-0.25 to -0.06)	0.002	−0.04	(-0.30 to 0.22)	0.767	0.420
Trail Making Test-5^a^	−0.40	(-0.62 to -0.18)	0.001	0.00	(-0.42 to 0.42)	0.998	0.094
MABC-2 total score^c^	0.15	(0.02 to 0.27)	0.024	0.00	(-0.29 to 0.28)	0.985	0.349
HiMAT total score^c,d^	0.36	(0.04 to 0.68)	0.026	0.04	(-0.78 to 0.85)	0.928	0.462

^a^Data missing for one VLBW participant with unilateral spastic cerebral palsy

^b^Mean score for both hands

^c^Data missing for one VLBW participant with bilateral spastic cerebral palsy

^d^Data missing for one control participant

^e^p-values for between-group differences in associations of physical and mental component summaries with motor skills

*HRQoL* = health-related quality of life, *VLBW* = very low birth weight, *CI* = confidence interval

*MABC-2* = Movement Assessment Battery for Children-2, *HiMAT* = High-level Mobility Assessment Tool

Higher scores on the Grooved Pegboard and Trail Making Test-5 indicate poorer function and higher scores on the MABC-2 and HiMAT indicate better function

Regression coefficient B for motor test, in linear regression with physical component summary and mental component summary as dependent variable, and motor test, group, and motor test x group as independent variables

## Discussion

In this study, VLBW young adults reported more mental health problems and lower HRQoL compared with controls at 23 years of age. In the VLBW group, mental health and HRQoL decreased from 20 to 23 years. Furthermore, in this group, more internalizing and total mental health problems as well as lower physical and mental HRQoL were associated with poorer performance on motor tests at 23 years, especially lower motor speed. When we excluded VLBW participants with CP and/or low IQ_est_, several group differences were no longer significant, but for the ASR, mean values and longitudinal changes were essentially the same, whereas associations with motor skills became weaker.

Strengths of this study are the longitudinal and multidisciplinary design and the use of reliable and valid methods [29, 31, 32]. However, sample size was limited, especially when we excluded participants with CP and/or low IQ_est_, resulting in reduced power in our analyses. Results and especially non-significant group differences should therefore be carefully interpreted, and one should focus more on means and standard deviations than *p*-values. Furthermore, our limited sample size did not give the possibility to study sub groups. Loss to follow-up may result in selection bias. The reason why 18 (33 %) of the invited VLBW young adults at 23 years did not want to participate and one did not fill out the questionnaires is not known. Our participants did not differ from non-participants on perinatal data or previous examinations of motor skills [20], mental health or HRQoL. The only difference in clinical characteristics between participants and non-participants was lower parental SES for VLBW non-participants. Low parental SES is associated with more mental health problems in childhood and adolescence [42], thus our results are more likely to be an underestimation than an overestimation of problems.

Self-report questionnaires like the ASR and SF-36 give participants the opportunity to describe their own perspective of their lives. However, self-reports are prone to social desirability bias, and cognitive function may influence the ability of self-perception and understanding questionnaires. We therefore performed analyses also when excluding VLBW participants with CP and/or low IQ_est_. In this VLBW cohort at 20 years, we found more symptoms of psychiatric disorders with diagnostic assessment by a psychiatrist than self-reported mental health problems on the ASR [43], and poorer executive functions on neuropsychiatric testing than on self-reports [44], which might indicate that the VLBW individuals underreported or had adjusted to their problems. Even though objective evaluations add valuable insights; how the young adults rate their own health, and especially their HRQoL, might be more important to them.

Our findings of more mental health problems in VLBW young adults, with emphasis on internalizing and attention problems are consistent with the literature [2, 9, 13, 45–47]. In our study, the VLBW young adults also had a tendency of reporting more anxious/depressed problems and social problems than the control group, supporting the suggested “Preterm behavioural phenotype” characterized by anxiety, inattention and social difficulties [4]. The VLBW young adults did not seem to be more depressed than controls according to BDI, in line with the findings of Räikkönen et al. [48]. However, Westrupp et al. [49] found that VLBW young adults in their late twenties were five times more likely to be diagnosed with depression. We speculate that depression may become more prevalent when our VLBW participants grow older. We also found that VLBW young adults reported less substance use with regard to alcohol, consistent with other studies [15, 50, 51], where some also describe less risk-taking behaviour [15, 18, 46]. These findings are in accordance with the personality type reported among young adults born with VLBW or very preterm (<33 weeks’ gestation), including less sensation seeking, extraversion and openness to experience, and higher conscientiousness, neuroticism and shyness [52, 53]. Even though increased parental monitoring and protectiveness cannot be excluded [47], Harrison [54] suggests that VLBW children and young adults have cognitive and behavioural deficits that isolate them from both their peers and their peers’ risk-taking behaviour, and that the isolation and withdrawal are caused by a lack of social and intellectual resilience. Cognitive function has been found to modify the risk of mental health problems of VLBW young adults in some studies [45]. In our study, group differences in mental health problems were no longer significant when we excluded VLBW participants with CP and/or low IQ_est_, however scores were essentially the same. It is of concern that the VLBW young adults scored significantly higher for critical items of clinically relevant psychiatric symptoms, also when we excluded participants with CP and/or low IQ_est_. We have previously reported a trend towards an increase of mental health problems from 14 to 20 years of age in this cohort [7], and the further increase from 20 to 23 years found in the current study is worrying and needs to be confirmed by other studies.

Findings on HRQoL in VLBW populations are conflicting. In contrast to our findings, the systematic reviews of Zwicker and Harris [11] and Allen et al. [9] concluded in four studies [12, 15, 18, 55] that VLBW young adults around age 20 have similar HRQoL to controls. However, poorer physical functioning [15] and lower objective quality of life [12] were reported. More recent studies from Switzerland [14] and New Zealand [16] also reported similar HRQoL to controls according to the SF-36 among young adults born with birth weight <1250 g or with VLBW at age 23. A Norwegian study by Båtsvik et al. [8] found lower scores for the SF-36 domains of bodily pain, vitality, social functioning, role-emotional and mental health among young adults born before week 28 or with a birth weight ≤1000 g compared with term-born controls at age 24. When they excluded participants with disabilities, group differences were significant for social functioning, role-emotional and mental health. When we excluded VLBW participants with CP and/or low IQ_est_ in our study, group differences on SF-36 were reduced and no longer significant, but mean scores where still lower for all domains in the VLBW group compared with the control group. Cooke [15] found poorer physical functioning on SF-36 in VLBW young adults able to attend mainstream schools, and Dinesen and Greisen [12] reported lower objective quality of life based on societal standards for VLBW individuals without disabilities at 18 years. Hence, both preterm born young adults with and without disabilities might be at risk for lower HRQoL than controls.

We speculate that the lower HRQoL among VLBW young adults found in our study could be partly due to challenges related to the transition to young adulthood. The underlying neuroimpairments of VLBW individuals may become more evident with these challenges, such as moving away from home, starting to study or work, finding a partner and living more independent and social lives [5]. In Norway, only 29 % of 20 to 24-year-olds live with their parents, in contrast to 83 % in Switzerland (Additional file 1), and parent-support might to some extent explain the discrepancy from the Swiss study [14]. In New Zealand, the proportion of 20 to 24-year-olds living with their parents was 32 % in 2006 (Additional file 2), similar to that in Norway. However, Darlow et al. [16] only used the component summaries of the SF-36 relative to 18 to 24-year norms, and their control group was recruited at 23 years among peers to the VLBW group. Our control group was followed from birth, and may therefore be more representative of the general population. The lower HRQoL found among extremely preterm young adults by Båtsvik et al. [8] supports our findings and might be more comparable as it is a Norwegian study. However, they studied young adults born before week 28 or with a birth weight ≤1000 g, a group that may be more vulnerable than VLBW individuals.

The increase in mental health problems found in our study may also have an impact on the reduction of HRQoL from 20 to 23 years in the VLBW group. In children and adolescents, HRQoL may decrease with time if mental health problems increase [56], and in VLBW young adults, internalizing problems are found to be strongly correlated to low HRQoL [57]. Longitudinal studies of HRQoL in VLBW populations are sparse and show few changes from adolescence to young adulthood. In one study, HRQoL from 14 to 19 years were stable in the VLBW group, however clinically important changes in psychological attributes of HRQoL were reported [57]. Van Lunenburg et al. [58] did not find any changes in HRQoL among VLBW young adults from 19 to 28 years of age. This may indicate that the lower HRQoL we found at age 23 may stabilize and improve over the next years. However, the methods and cultural settings in these studies are not directly comparable. Both the VLBW and control group in our study reported a reduction in general health from 20 to 23 years, which might be a general change during this life period. People’s evaluations of their general health are found to be dynamic and changing within a two-year period for half the adult population [59]. More studies are needed to understand the changes in HRQoL in preterm populations with time.

We have previously reported that VLBW young adults had poorer motor skills than controls at 23 years [20]. The current study shows that internalizing and total mental health problems as well as lower physical and mental HRQoL were associated with poorer motor skills, especially motor speed, also when we excluded VLBW participants with CP and/or low IQ_est_. Studies of adults with developmental coordination disorder showed that they reported more symptoms of anxiety and depression [60] and lower quality of life than peers [22]. Both among adults with normal birth weight and <1000 g, self-reported childhood coordination problems were associated with elevated levels of inattention and symptoms of anxiety and depression [21]. Our study confirms and extends the existing knowledge by using clinically assessed motor skills in young adulthood to establish the associations of mental health and HRQoL with motor skills in VLBW young adults.

There is reason to believe that the motor and mental health problems in preterm populations share a common cause. The preterm brain is susceptible to a cascade of adverse events, often referred to as the ‘Encephalopathy of prematurity’ [1], likely to affect both motor skills and mental health. The altered brain development continues into young adulthood [61, 62], and we have previously reported changes in brain white matter that were associated with cognitive, motor and mental health impairments among VLBW adolescents [63]. Poor executive functioning with difficulties holding information in mind and switching between mental sets is related to behavioural functioning [2], and might also partly explain the social difficulties of VLBW individuals. There is emerging evidence that stressful prenatal and neonatal factors, such as preterm birth, may imprint a pattern of physiological activity in the developing brain, known as “foetal programming” [64]. The immune system and the hypothalamus-pituitary-adrenal stress-regulating system are found to be especially vulnerable to long-term alteration in children born preterm [65]. We therefore speculate that an altered stress-response might contribute to more mental health problems among the VLBW young adults.

### Clinical implications

This study adds to the knowledge of mental health and HRQoL in VLBW young adults. In HRQoL studies, a difference or change of 0.5SD is suggested to reflect a clinical important difference [66], and the finding of more mental health problems and lower HRQoL is therefore likely to impact the daily life of these young adults. The reduction of mental health and HRQoL in the transition to adulthood emphasizes the importance of long-term follow-up and performing longitudinal analyses. Awareness of the association of mental health and HRQoL with motor skills may be important as motor problems are easily identified in early childhood. This makes selection for early intervention possible. There is reason to be observant of and encourage research on problems that might become more visible as the VLBW young adults enter independent living and encounter the demands of adulthood.

## Conclusions

In conclusion, VLBW young adults reported poorer and declining mental health and HRQoL in the transitional phase into adulthood compared with matched term-born controls. They seemed to have a cautious lifestyle with more internalizing problems and less alcohol use. Poorer motor skills were associated with more mental health problems and especially lower physical HRQoL, the cause of which is likely to be of shared aetiology.

## Additional files

Additional file 1: Share of young people living with their parents in Europe. (XLS 30 kb) Additional file 2: Proportion (%) of young people living with their parents in New Zealand, 1981–2006. (DOCX 14 kb)

## Abbreviations

ASR: Adult Self-Report
BDI: Beck Depression Inventory
CI: confidence interval
CP: cerebral palsy
GMFCS: Gross Motor Function Classification System
GP: Grooved Pegboard Test
HiMAT: High-Level Mobility Assessment Tool
HRQoL: health-related quality of life
IQ_est_: estimated intelligence quotient
Movement ABC-2: Movement Assessment Battery for Children-2
NICU: neonatal intensive care unit
SD: standard deviation
SES: socioeconomic status
SF-36: Short Form 36 Health Survey
VLBW: very low birth weight
