# Supplementary material for: Long-term follow-up of mental health, health-related quality of life and associations with motor skills in young adults born preterm with very low birth weight
Source: Health Qual Life Outcomes. 2016 Apr 7;14:56. doi: 10.1186/s12955-016-0458-y (PMC4823914; doi:10.1186/s12955-016-0458-y)
Supplement: Additional file 2: — Proportion (%) of young people living with their parents in New Zealand, 1981–2006. (DOCX 14 kb) [file 12955_2016_458_MOESM2_ESM.docx]

**Additional file 2:** Proportion (%) of young people living with their parents in New Zealand, 1981–2006

| **Census year** | **15–19 years** | **20–24 years** | **15–24 years** |
| --- | --- | --- | --- |
|  |  |  |  |
| 1981 | 71 | 28 | 51 |
| 1986 | 77 | 31 | 54 |
| 1991 | 77 | 36 | 57 |
| 1996 | 76 | 35 | 55 |
| 2001 | 76 | 32 | 55 |
| 2006 | 79 | **32** | 56 |
|  |  |  |  |

*Source: 1981, 1991: Statistics New Zealand (1994) p 59-60;*

*1986, 1996, 2001, 2006: Statistics New Zealand, unpublished census data*
